# Supplementary material for: Influenza H1 Mosaic Hemagglutinin Vaccine Induces Broad Immunity and Protection in Mice
Source: Vaccines (Basel). 2019 Nov 23;7(4):195. doi: 10.3390/vaccines7040195 (PMC6963302; doi:10.3390/vaccines7040195)
Supplement: Supplementary file 1 [file vaccines-07-00195-s001.pdf]

Supplemental Figure S1
Western blot analysis of recombinant HA expressed in Adenovirus type 5. Relative protein quantity, as determined by densitometry, is shown for each band.

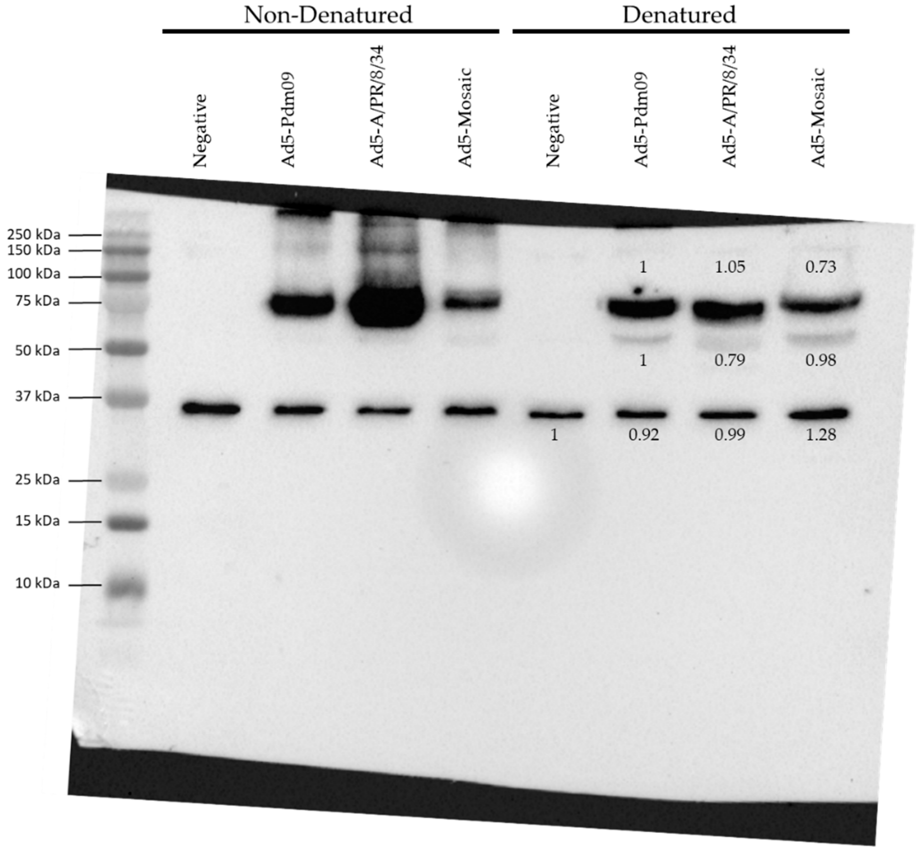

Supplemental Table 1. Overview of Immunostimulatory Peptides Mapped by ELISpot Assay. The Immune Epitope Database and Analysis Resource (iedb.org) from the NIAID was used to search all immunostimulatory peptides identified by IFN-γ ELISpot Assay with the following parameters; Epitope: linear epitope, substring; Antigen Organism: any; Host: any; Assay: positive assays only; MHC Restriction: any MHC restriction; Disease: any disease. The peptide number, sequence, and vaccine groups for each positive peptide are shown. Epitopes within the peptide sequence that have induced immune responses by binding MHC, binding B cells, inducing cytokines, inducing cytotoxicity, or other relevant responses are specified.

	Peptide	Peptide Sequence	Positive Vaccines	Positive MHC	B cell Binding	T cell cytokines	T cell cytotoxicity	Other
A/Brisbane/59/2007	31	YANNKEKEV LVLWGVHH	Mosaic	Mamu-A1	Yes	-	-	-
	36	ENAYVSVVSS HYSRKFT	Mosaic	HLA-A; HLA-B	Yes	-	-	-
	37	VVSSHYSRKF TPEIAKR	Mosaic	-	-	-	-	-
	77	NVKNLYEKV KSQLKNNA	Mosaic, Pdm09, A/PR/8/34	H2-Kd, H2-Db, H2-Kb, H2-Ld, HLA-A	Yes	IFN-γ; IL-4	Yes	-

	89	ILAIYSTVASS LVLLVS	Mosaic, Pdm09, A/PR/8/34	H2-Kd; H2-Kb; HLA-B37; HLA-A; Mamu-A1	yes	IFN-γ; IL-4; IL-2; IL-13; TNFα	yes	Decreased disease; degranulation; T cell binding & proliferation
A/NC/20/1999	31	SYVNNKEKE VLVLWGVH	Mosaic	Mamu-A1	-	-	-	-
	36	HTENAYVSV VSSHYSRR	Mosaic	HLA-A; HLA-B	yes	-	-	-
	78	VKNLYEKVK SQLKNNAK	Mosaic, Pdm09, A/PR/8/34	H2-Kd; H2-Db; H2-Kb	yes	IFN-γ; IL-4	yes	-
	90	YQILAIYSTV ASSLVLL	Mosaic, Pdm09, A/PR/8/34	H2-Kd; H2-Kb; HLA-B37; HLA-A; Mamu-A1	yes	IFN-γ; IL-4; IL-2; IL-13; TNFα	yes	Decreased disease; degranulation; T cell binding & proliferation
A/CA/07/2009	38	VTAACPHAG AKSFYK	Pdm09	HLA-A	-	-	-	-
	39	CPHAGAKSF YKNLIW	Pdm09	HLA-A	-	-	-	-
	40	GAKSFYKNLI WLVKK	Pdm09	HLA-A	-	-	-	-
	43	VKKGNSYPK LSKSYI	Pdm09	-	-	-	-	-
	44	NSYPKLSKSYI NDKG	Pdm09	-	-	-	-	-
	47	DKGKEVLVL WGIHHP	Mosaic	-	yes	-	-	-
	54	DAYVFVGSSR YSKKF	Pdm09	-	-	-	-	T cell quantitative binding
	115	SNVKNLYEK VRSQLK	Mosaic, Pdm09, A/PR/8/34	H2-Db; H2-Kb; SLA-3	-	-	-	-
	116	NLYEKVRSQL KNNAK	Pdm09, A/PR/8/34	SLA-3	-	-	-	-
	133	QILAIYSTVAS SLVL	Mosaic, Pdm09, A/PR/8/34	H2-Kd; H2-Kb; HLA-B37; HLA-A; Mamu-A1	yes	IFN-γ; IL-4; IL-2; IL-13; TNFα	yes	Decreased disease; degranulation; T cell binding & proliferation
	135	VASSLVLVVS LGAIS	Mosaic, Pdm09, A/PR/8/34	-	-	-	-	-
